# Supplementary material for: Friendly-rivalry solution to the iterated n-person public-goods game
Source: PLoS Comput Biol. 2021 Jan 21;17(1):e1008217. doi: 10.1371/journal.pcbi.1008217 (PMC7853487; doi:10.1371/journal.pcbi.1008217)
Supplement: S1 Appendix — (PDF) [file pcbi.1008217.s001.pdf]

Supporting Information

Friendly-rivalry solution to the iterated n-person public-goods game

Yohsuke Murase
RIKEN Center for Computational Science, Kobe, Hyogo 650-0047, Japan

Seung Ki Baek
Department of Physics, Pukyong National University, Busan 48513, Korea

December 30, 2020

Supporting information

S1 Appendix. Computational check for efficiency, defensibility, and distinguishability

Efficiency

The most direct method for checking efficiency is to construct a Markovian transition matrix. For example, if we check efficiency of WSLS, the matrix can be written as

$$
h_t \begin{array}{c} \\ (c,c) \\ (c,d) \\ (d,c) \\ (d,d) \end{array}
\overset{\displaystyle h_{t-1}}{\begin{array}{cccc} (c,c) & (c,d) & (d,c) & (d,d) \end{array}}
\begin{bmatrix}
(1-\epsilon)^2 & \epsilon^2 & \epsilon^2 & (1-\epsilon)^2 \\
\epsilon(1-\epsilon) & \epsilon(1-\epsilon) & \epsilon(1-\epsilon) & \epsilon(1-\epsilon) \\
\epsilon(1-\epsilon) & \epsilon(1-\epsilon) & \epsilon(1-\epsilon) & \epsilon(1-\epsilon) \\
\epsilon^2 & (1-\epsilon)^2 & (1-\epsilon)^2 & \epsilon^2
\end{bmatrix}, \tag{S1}
$$

where each element is the conditional probability to observe $h_t = (A_t, B_t)$ for given $h_{t-1} = (A_{t-1}, B_{t-1})$. The stationary probability distribution of the above process can be calculated explicitly if m is small [1], but it is often easier to obtain a numerical solution, e.g., by using the QR algorithm [2]. If the stationary probability of full cooperation approaches 100% as ϵ decreases, the strategy is judged as efficient.

Note that the above direct method calculates the effect of every possible type of error all at once. A quicker way is to look at the connection structure of history profiles [3], and let us only outline the idea briefly: In the above example of WSLS, suppose that $\epsilon = 0$ as the zeroth-order approximation of the actual process. It is then immediately clear that from every initial condition the players should eventually end up with (c, c) and stay there. Once this is the case, we can prove that the existence of $\epsilon \ll 1$ perturbs the stationary distribution only to a marginal degree so that we will actually get the same answer as $\epsilon \to 0^+$. Or, if the zeroth-order approximation fails to give such a definite answer, then we have to modify the connection structure to the first order by including transitions with probability of $O(\epsilon)$. This process is repeated with increasing the order of ϵ one by one, until we reach the answer. One may refer to Ref. [3] for the details.

Defensibility

When a focal player's strategy is given, his or her action is determined at every history profile, whereas the other $(n-1)$ co-players' are not. Therefore, each history profile can lead to 2^{n-1} possible history profiles in the next round. If each history profile is regarded as a node, we thus obtain a connection structure in which every node has 2^{n-1} outward links. At every node, we can see the focal player's instantaneous payoff based on the actions taken in round t. The point is that we are concerned about the focal player's long-term average payoff, to which only results from cyclic loops can contribute. The basic idea for checking defensibility is thus to find all the loops in the connection structure: If a loop puts the focal player at a payoff disadvantage compared with the co-players, let us call it a negative loop. If the connection structure contains no such negative loops, the corresponding strategy is defensible because it is impossible to make the player's long-term average payoff lower than the co-players' no matter what they choose. To detect all the negative loops, we have used the Floyd-Warshall algorithm [4, 5, 3].

Distinguishability

The distinguishability criterion can be checked in a similar way to the one for the efficiency criterion because it is essentially determined from the stationary distribution. For example, we can construct a transition matrix between a given strategy and AllC as in Eq. (S1). For the strategy to be distinguishable, the stationary distribution must keep a finite amount of probability to observe defection against AllC as $\epsilon \to 0^+$.

References

[1] T. You, M. Kwon, H.-H. Jo, W.-S. Jung, S. K. Baek, Chaos and unpredictability in evolution of cooperation in continuous time, Phys. Rev. E 96 (6) (2017) 062310.

[2] M. E. J. Newman, Computational Physics, CreateSpace Independent, San Bernardino, CA, 2013.

[3] Y. Murase, S. K. Baek, Five rules for friendly rivalry in direct reciprocity, Sci. Rep. 10 (2020) 16904.

[4] S. Hougardy, The Floyd–Warshall algorithm on graphs with negative cycles, Inf. Process. Lett. 110 (8-9) (2010) 279–281.

[5] Y. Murase, S. K. Baek, Seven rules to avoid the tragedy of the commons, J. Theor. Biol. 449 (2018) 94–102.
