# Supplementary material for: Friendly-rivalry solution to the iterated n-person public-goods game
Source: PLoS Comput Biol. 2021 Jan 21;17(1):e1008217. doi: 10.1371/journal.pcbi.1008217 (PMC7853487; doi:10.1371/journal.pcbi.1008217)
Supplement: S1 Table — (PDF) [file pcbi.1008217.s002.pdf]

Supporting Information

Friendly-rivalry solution to the iterated n-person public-goods game

Yohsuke Murase
RIKEN Center for Computational Science, Kobe, Hyogo 650-0047, Japan

Seung Ki Baek
Department of Physics, Pukyong National University, Busan 48513, Korea

December 30, 2020

Supporting information

Table S1: **Summary of mathematical symbols used in this work.**

n	number of players
m	memory length
t	time in units of rounds
ρ	multiplication factor of the public-goods game
ϵ	probability of implementation error
n_c	number of cooperators
X_t	player X's action at t
h_t	history profile composed of the players' actions over the past m rounds
$\pi_X^{(t)}$	player X's instantaneous payoff in round t
Π_X	player X's long-term average payoff
$\Delta_A^{\tau_1,\tau_2}$	Alice's number of defections during a time interval $[\tau_1, \tau_2]$
t^*	the last round of full cooperation
N_d	maximum difference in numbers of defections in memory
N	population size in an evolutionary game
$P_{j,j\pm1}$	probability that the number of mutants increases or decreases from j by one
Γ_j	$P_{j,j-1}/P_{j,j+1}$
$f_{x\to y}$	probability that a player changes the strategy from x to y
σ	strength of selection
ϕ_{xy}	fixation probability of mutant x in a resident y-population
$s_{\alpha\beta}$	α's long-term payoff against β in the two-person PG game
$s_{\alpha\beta\gamma}$	α's long-term payoff against β and γ in the three-person PG game
s_x	average payoff of x
b	benefit of cooperation in the donation game
$p_{\mu\nu}$	probability to cooperate when two players did μ and ν, respectively

S1 Table.
