# Supplementary material for: Friendly-rivalry solution to the iterated n-person public-goods game
Source: PLoS Comput Biol. 2021 Jan 21;17(1):e1008217. doi: 10.1371/journal.pcbi.1008217 (PMC7853487; doi:10.1371/journal.pcbi.1008217)
Supplement: Attachment — Submitted filename: Review_PLOS_CAPRI.pdf [file pcbi.1008217.s003.pdf]

The authors present results on the behavior of a specific class of strategy, which they call CAPRI-n, in the iterated n player public goods game. The strategy has memory $2n-1$ and is constructed so as to ensure 1) mutual cooperation in the absence of execution errors (efficiency) 2) that no alternate strategy Y can gain a higher payoff against CAPRI-n than CAPRI-n does against Y (defensibility) and 3) that it can exploit mutual cooperation, ALLC (distinguishability). This is an interesting topic and the strategies presented are, to my knowledge, novel, but I have several concerns about the completeness of the analysis presented in the current version of the manuscript.

1. **Stationary distribution of payoffs for CAPRI-n against self**. As the authors note, a game played between a group of n players using this strategy, in the limit of small errors $e \to 0$, has two stationary states: mutual cooperation and mutual defection. By construction, the only way to recover mutual cooperation from a state of mutual defection is if $n-1$ players simultaneously cooperate in a given round, which occurs with probability $\pi_{d\to c} = e^{n-1}$. On the other hand, a state of mutual defection can be reached from a state of mutual cooperation if at least one player commits an error during each of $2n-1$ successive rounds, such that mutual cooperation does not occur within any player's memory. While a complete analysis of the probability of this transition requires us to account for cases in which mutual cooperation is inadvertently restored through errors, we can estimate the probability as $\pi_{c\to d} \approx (1-(1-e)^n)^{2n-1}$ where $(1-e)^n$ is the probability that nobody makes an error in a given round, meaning $(1-(1-e)^n)$ is the probability that *someone* makes an error in a given round. If we take the limit of small e and Taylor expand this expression we recover $\pi_{c\to d} \approx n^{2n-1}e^{2n-1}$. A simple criterion for a strategy to be good at enforcing cooperation is $\pi_{d\to c} > \pi_{c\to d}$ which occurs if $n^{2n-1}e^n < 1$. If we rearrange this expression and neglect terms in $1/n$ this inequality can be reduced to $n < 1/\sqrt{e}$. i.e. when the game size is greater than roughly $1/\sqrt{e}$ we expect CAPRI-n to do a bad job of enforcing mutual cooperation with itself. I have done this analysis quickly but I hope the authors see that this is an issue that must be addressed.

2. **Evolutionary robustness.** The authors do not, as far as I can see, provide any proof of evolutionary robustness. To recap, a strategy is evolutionary robust if the probability of any new strategy reaching fixation when introduced to a population where the focal strategy is resident is less than $1/N$ where N is the population size. It is true that if there are zero errors CAPRI-n is robust by construction, but as soon as errors are non-zero, the expected payoff for a population of CAPRI-n players is reduced below that for mutual cooperation due to the possibility of quasi-stable mutual defection, as discussed above. Thus the probability of any new strategy reaching fixation in a population of CAPRI-n depends not only on how that strategy performs agains CAPRI-n but also how it performs in groups composed only of itself. I cannot find any discussion of this issue in the manuscript.

3. **Memory errors.** CAPRI-n is constructed specifically to deal with execution errors by players who wish to cooperate, but to do this it requires a memory off length $2n-1$. As memory length increases, it is reasonable to also consider the effect of memory errors. It is not clear to me how well CAPRI-n will deal with these (again see discussion above).

4. **Distinguishability.** The need for a strategy to be able to exploit ALLC is not clearly motivated since, as the authors note (line 303-305) CAPRI-n can be neutrally invaded by other simple memory-1 strategies.

5. **Simulations.** Simulations are performed in which CAPRI-n is competed against deterministic memory-1 strategies. This is not comparing like with like. The comparison should be with other deterministic memory $2n-1$ strategies.
