# Supplementary material for: Friendly-rivalry solution to the iterated n-person public-goods game
Source: PLoS Comput Biol. 2021 Jan 21;17(1):e1008217. doi: 10.1371/journal.pcbi.1008217 (PMC7853487; doi:10.1371/journal.pcbi.1008217)
Supplement: Attachment — Submitted filename: response.pdf [file pcbi.1008217.s004.pdf]

Pukyong National University
Department of Physics
Busan 48513, Korea
SEUNG KI BAEK
Email: seungki@pknu.ac.kr
Tel: +82 51 629 5576
Fax: +82 51 629 5549

November 6, 2020

Dear Editor,

First of all, we would like to thank you and all the reviewers who carefully read our manuscript and gave us constructive comments. We are glad to hear that they found our manuscript "very interesting", "well presented", and "novel". We have tried our best to revise our manuscript in accordance with their comments. The manuscript has been substantially improved thanks to their advice, and we hope that it is now suitable for publication in PLOS Computational Biology.

Yours Sincerely,

Yohsuke Murase & Seung Ki Baek

To Editor

> **Editor:** The paper has now been revised by three of our reviewers, who have made a number of criticisms that are sufficiently adverse as to suggest that a major revision is due before a final decision about publication can be made. We therefore ask you to address all comments by the reviewers. However, we ask you to pay particular attention to:
> - the technical aspects raised by reviewer 3. This might require to show that the results are robust beyond what is now presented in the MS.

Answer: We have tried our best to answer Reviewer 3's comments as will be detailed below.

> **Editor:** - We would also like that similarities and differences with respect to the paper arXiv:2004.00261 were clearly stated. PCB only published highly original contributions, and we appreciate that you have studied the same strategy for the PD game, which apparently leads to very similar findings and even the discussion presented.

Answer: At the end of Introduction, we have clearly stated that this work is an n-person generalization of arXiv:2004.00261 (now published in Scientific Reports), which concerned only the iterated prisoner's dilemma game (**List of changes #1**).

To Reviewer #1

> **Reviewer:** I found the study "Friendly-rivalry solution to the iterated n-person public-goods game" by Murase and Baek very interesting, if not important. I believe that the study should be published subject to addressing the issues listed below.

Answer: We are grateful for your helpful comments. We are pleased to see that you also found our manuscript both interesting and important.

> **Reviewer:** NON-NEGOTIABLE
> * There are many mathematical symbols. A table of symbols is absolutely needed.

Answer: We have added a table as Supporting Information. In addition, we have also revised the manuscript to reduce the number of symbols (**List of changes #2**).

> **Reviewer:** MAJOR
> L209-225... I really struggled trying to understand the argument about efficiency. If I had more time, maybe I would fully grasp it, but as it is, the available information did not really convince me. I strongly suggest that the authors consider rephrasing the argument for the benefit of their readers.

Answer: We have elaborated on how the strategy satisfies the efficiency criterion. Please take a look at pages 7 and 8 of this revised manuscript (**List of changes #3**).

> **Reviewer:** L265-272... This whole paragraph sounds as if the authors were saying: "We checked our claims and you should trust us!" It is utterly unclear whether the checks were performed on paper or computationally. If the former is the case, then the authors should detail their calculations in an appendix or supporting text. If the latter is the case, then a reference to the precise part of the code should be made available.

Answer: We have computationally checked the criteria. An SI appendix has been newly added to explain the basic ideas behind the algorithms, and the codes are available at a link in Acknowledgment (**List of changes #4**).

> **Reviewer:** Fig. 3... What do gray dots represent? What does blueish color coding stand for?

Answer: We have removed the gray dots, which appeared for no particular reason while we generated the figure in the PDF format. The darker shades toward blue indicate higher frequency of occurrence (**List of changes #5**).

> **Reviewer:** MINOR
> L139... Eq. (1) does not really define a matrix.

Answer: We have rephrased it as "payoff definition" (**List of changes #6**).

> **Reviewer:** L191, 192... I'm not sure if I got 'former' and 'latter' right at this particular spot in the manuscript. Perhaps the authors should rephrase.

Answer: Thank you for the comment. We have revised the expression to avoid any possible confusion (**List of changes #7**).

> **Reviewer:** L266... CAPRI-2 rather than CAPRI-n.

Answer: Thank you again. We have fixed it (**List of changes #8**).

> **Reviewer:** L366... The authors should cite OACIS in the Methods section.

Answer: We have now cited it at the end of Materials and Methods (**List of changes #9**).

To Reviewer #2

> **Reviewer:** This manuscript presents an interesting study on the social dilemma of cooperation by an approach based on the iterated n-person public-goods Game. The manuscript is well presented. I found correct mathematics as well as the results obtained both via simulations and formal reasoning. However, I have some concerns about both the justification of the model and the discussion.

Answer: We are grateful for your review comments. We are very pleased to hear that you found our work "interesting", "correct", and "well presented."

> **Reviewer:** The authors extend the concept of Nash equilibrium to long-term payoffs. This extension has been adopted in recent literature, for example, as authors cited, in the context of zero-determinant strategies. The authors define a set of conditions for a particular collaborative strategy (CAPRI). If I understand the model correctly, the condition for the Nash equilibrium, for the particular case of a symmetric strategic profile where all the agents share the strategy Ω, is reformulated as: "It must be guaranteed that none of the co-players can obtain higher long-term payoffs against Ω regardless of their strategies and initial states when $e = 0$."
> Furthermore, the required memory length of players adopting such a strategy is $m = 2n - 1$. I find this concept far from the original Nash Equilibrium, and, at least, would expect a deep discussion by the authors on this (or perhaps to rename it).

Answer: Thank you for giving us a chance to clarify an important point. We may regard an n-person game as a machine that receives n strategies as inputs and then returns a list of n payoffs as the output. If, by the rules of the game, the payoffs happen to be the same as long-term averages in the iterated n-person public-goods game, we are saying that there exists a strategy such that if everyone is using it, no one can increase his or her own payoff by submitting a different strategy to the machine. We thus believe that the concept of Nash equilibrium still applies here in its full sense.

One possible reason of confusion might be that our original manuscript just referred readers to our previous papers for the connection between our criteria and the notion of Nash equilibrium with no explicit explanation. What we meant was that our criteria constitute a sufficient condition to prove the strategy as forming a Nash equilibrium. We have elaborated on this connection on page 4 in this revised manuscript (**List of changes #10**).

> **Reviewer:** Nevertheless, the authors study the system for small values of n, which, one the one hand, make the memory requirement realistic while, on the other hand, bring this approach very close to that of pairwise interactions which has been addressed by the authors in Ref. 22.
> The memory considered $(2n - 1)$ is very long for large sets of co-players, furthermore when the authors state "not far from human behavior." Does it mean that, in an n-agents interaction, an agent may have a memory of $2n - 1$ previous interactions of everybody? I think that the authors should contextualize their model and justify it according to its particular requirements on memory length to differentiate it from that of pairwise interactions.

Answer: Our main goal has been to modify the result of our previous work and thereby generalize it to the n-person game. As has been explained on page 3, it is usually difficult to find an n-person generalization of a set of successful rules that are devised to solve a two-person game. As you correctly pointed out, we have checked small values of $n \leq 4$, but this is intended as a 'proof of concept', and the principle itself remains valid for $n > 4$ as well.

An important point is that a player does not necessarily need the exact history: To be a friendly rival, one only has to remember t^*, the last time of mutual cooperation, and how many times the players have defected since t^*. This means that the actual cognitive burden is not as demanding as it might sound at first. In the last paragraph of Discussion, for example, we are referring to an experimental report that a player's reputation is mainly composed of his or her last action and the average frequency of defection, which suggests that people may be apt to do such a task.

To Reviewer #3

> **Reviewer:** The authors present results on the behavior of a specific class of strategy, which they call CAPRI-n, in the iterated n player public goods game. The strategy has memory $2n1$ and is constructed so as to ensure 1) mutual cooperation in the absence of execution errors (efficiency) 2) that no alternate strategy Y can gain a higher payoff against CAPRI-n than CAPRI-n does against Y (defensibility) and 3) that it can exploit mutual cooperation, ALLC (distinguishability). This is an interesting topic and the strategies presented are, to my knowledge, novel, but I have several concerns about the completeness of the analysis presented in the current version of the manuscript.

Answer: We are grateful for your careful reading and constructive comments. We have tried our best to revise our manuscript according to the comments.

> **Reviewer:** 1. Stationary distribution of payoffs for CAPRI-n against self.
> As the authors note, a game played between a group of n players using this strategy, in the limit of small errors $e \to 0$, has two stationary states: mutual cooperation and mutual defection. By construction, the only way to recover mutual cooperation from a state of mutual defection is if $n-1$ players simultaneously cooperate in a given round, which occurs with probability $\pi_{d\to c} = e^{n-1}$. On the other hand, a state of mutual defection can be reached from a state of mutual cooperation if at least one player commits an error during each of $2n-1$ successive rounds, such that mutual cooperation does not occur within any player's memory. While a complete analysis of the probability of this transition requires us to account for cases in which mutual cooperation is inadvertently restored through errors, we can estimate the probability as $\pi_{c\to d} = (1-(1-e)^n)^{2n-1}$ where $(1-e)^n$ is the probability that nobody makes an error in a given round, meaning $(1-(1-e)^n)$ is the probability that someone makes an error in a given round. If we take the limit of small e and Taylor expand this expression we recover $\pi_{c\to d} = n^{2n-1}e^{2n-1}$. A simple criterion for a strategy to be good at enforcing cooperation is $\pi_{d\to c} > \pi_{c\to d}$ occurs if $n^{2n-1}e^{n} < 1$. If we rearrange this expression and neglect terms in $1/n$ this inequality can be reduced to $n < 1/\sqrt{e}$. i.e. when the game size is greater than roughly $1/\sqrt{e}$ we expect CAPRI-n to do a bad job of enforcing mutual cooperation with itself. I have done this analysis quickly but I hope the authors see that this is an issue that must be addressed.

Answer: This is an excellent comment. In addition to the orders of ϵ, we also have to consider the prefactors if ϵ takes a finite value, and the prefactor of $\pi_{c \to d}$ is likely to increase faster than that of $\pi_{d \to c}$ as n grows. If this is the case, it will limit the applicability of CAPRI-n for a large n in reality. We have added a paragraph to discuss this issue to the last section (**List of changes #11**).

> **Reviewer:** 2. Evolutionary robustness.
> The authors do not, as far as I can see, provide any proof of evolutionary robustness. To recap, a strategy is evolutionary robust if the probability of any new strategy reaching fixation when introduced to a population where the focal strategy is resident is less than $1/N$ where N is the population size. It is true that if there are zero errors CAPRI-n is robust by construction, but as soon as errors are non-zero, the expected payoff for a population of CAPRI-n players is reduced below that for mutual cooperation due to the possibility of quasi-stable mutual defection, as discussed above. Thus the probability of any new strategy reaching fixation in a population of CAPRI-n depends not only on how that strategy performs against CAPRI-n but also how it performs in groups composed only of itself. I cannot find any discussion of this issue in the manuscript.

Answer: This is another excellent comment. We can prove that a friendly rival always has evolutionary robustness when $n = 2$ and $\epsilon \to 0$, and it is now stated below Eq. (15) in this revised manuscript. For $n > 2$, on the other hand, we have no proof for evolutionary robustness, and we made this point clear by adding sentences below Eq. (16). Overall, our original usage of "evolutionary robustness" was misleading, so we have corrected the expression throughout the manuscript (**List of changes #12**).

> **Reviewer:** 3. Memory errors.
> CAPRI-n is constructed specifically to deal with execution errors by players who wish to cooperate, but to do this it requires a memory off length $2n - 1$. As memory length increases, it is reasonable to also consider the effect of memory errors. It is not clear to me how well CAPRI-n will deal with these (again see discussion above).

Answer: We believe that we can make basically the same argument as the one on perception error in the second paragraph of Discussion. There, we have written as follows: *"Although we have considered only implementation error, perception error can also be corrected if it occurs with sufficiently low probability: The disagreement between the players history profiles due to the perception error will soon be removed at full defection, and the players will escape from mutual defection with probability of $O(\epsilon^n)$. Unless another perception error perturbs this process, the players will eventually arrive at full cooperation, overcoming the perception error."* Although this is a rather weak statement, this seems to be the best we can do at the moment.

Reviewer: 4. Distinguishability.
The need for a strategy to be able to exploit ALLC is not clearly motivated since, as the authors note (line 303-305) CAPRI-n can be neutrally invaded by other simple memory-1 strategies.

Answer: It is well known in the literature that neutral drift of AllC eventually causes the collapse of cooperation. This is the reason that we check distinguishability from AllC in the hope of removing the weakest spot at least. We agree that it would be great if our strategy had distinguishability with respect to other non-defensible strategies as well, but we are unaware of any systematic method to check such an extended version of distinguishability within reasonable time. The I-rule of CAPRI-n could be regarded as a very crude way to slow down the invasion of naive cooperators by prescribing d almost all the time.

Reviewer: 5. Simulations.
Simulations are performed in which CAPRI-n is competed against deterministic memory-1 strategies. This is not comparing like with like. The comparison should be with other deterministic memory $2n-1$ strategies.

Answer: We have conducted additional simulation to check the performance of CAPRI-n against deterministic strategies with the same memory length and depicted the results in Fig. 6 (**List of changes #13**). We can sample only a tiny fraction of such strategies, but as far as we can tell, their fixation probabilities are all lower than or equal to that of a neutral mutant. Interestingly, it seems more difficult for a randomly sampled strategy to achieve fixation in the three-person game although there exists no proof for evolutionary robustness in this case.

List of changes

#1 At the end of Introduction, we have added the following sentence:

> In this way, this work modifies and generalizes our previous finding of $n = 2$ and $m = 3$, i.e., a memory-three friendly-rivalry strategy for the iterated PD game.

#2 Table S1 has been added as Supporting Information on Page 17. We have also simplified the mathematical description on Page 5 to reduce the number of symbols.

#3 We have expanded the explanation of efficiency on pages 7 and 8 by using several examples.

#4 At the beginning of Results, we have now written:

> To check the validity of our construction, we computationally examined the three criteria by using graph-theoretic calculations used in [19, 21, 34]. For $n = 2$, we directly confirmed that CAPRI-2 is indeed a successful strategy satisfying all the three criteria. For $n = 3$, we conducted mapping to an automaton to obtain a simplified yet equivalent graph representation to reduce computational complexity, and our graph-theoretic calculation confirmed that the resulting automaton indeed passed all the criteria. ... See S1 Appendix for more discussion on computational details.

A newly added SI appendix on page 16 explains the basic ideas of our computational approaches.

#5 Figure 3 has been replaced by a new one without the gray dots. The caption has also been revised as follows:

> Distribution of long-term payoffs when a CAPRI-n player meets co-players whose $p_{\mu\nu}$'s are randomly sampled from the unit interval. Darker shades toward blue indicate higher frequency of occurrence. The multiplication factors for $n = 2$, 3, and 4 are 1.5, 2, and 3, respectively, and the solid lines indicate the region of feasible payoffs. In each case, the filled circle means the long-term payoffs when CAPRI-n is adopted by all the players, whereas the cross shows those of TFT players as a reference point. In each panel, we have drawn a dotted line along the diagonal as a simple check for defensibility. For $n = 3$ or 4, the parallelogram surrounding the blue area indicates the set of feasible payoffs when the focal player is AllD, which indicates that the behavior of CAPRI-n is similar to AllD against most of the memory-one players.

#6 In L144, we have written:

According to payoff definition [Eq. (1)], ….

#7 From L200 to L202, we have now clarified the expression as follows:

> The former case, i.e. the case when she comes back to C via A, means that Alice has already been compensated for the payoff loss: Otherwise, she would not have state A. In the latter case when C is accessed via R, on the other hand, the only possible history profile is (*ddddd*; *ddddc*; *ddddc*) unless she made a mistake, which means the compensation has been done in the last round.

#8 In L314 and L315, we have changed CAPRI-n to CAPRI-2:

> For $n = 2$, we directly confirmed that CAPRI-2 is indeed a successful strategy satisfying all the three criteria.

#9 We have cited OACIS at the end of Materials and Methods in L310.

#10 On page 4, we have clearly mentioned the connection between the criteria and the concept of Nash equilibrium:

> A symmetric strategy profile which consists of a friendly-rivalry strategy forms a cooperative Nash equilibrium, and the proof is straightforward: Assume that everyone initially uses a friendly-rivalry strategy Ω with earning ρ per round. If one player, say, X, changes his or her strategy alone, X's payoff will change to Π_X, while each of the co-players earns Π_Ω. Defensibility guarantees that $\Pi_X \le \Pi_\Omega$, and full cooperation is Pareto-optimal, i.e., $(n-1)\Pi_\Omega + \Pi_X \le n\rho$. Combining these two inequalities, we see that
>
> $$(n-1)\Pi_X + \Pi_X \le (n-1)\Pi_\Omega + \Pi_X \le n\rho,$$
>
> which means that $\Pi_X \le \rho$. The player cannot increase his or her payoff by deviating from Ω alone.

#11 In Discussion, we have newly added the following paragraph:

We have assumed the small-ϵ limit, but an important question is how the performance of CAPRI-n will be affected if ϵ takes a finite value. One possibility is that it could set a limit on the maximum number of players in regard to the efficiency criterion: The transition probability from full defection to cooperation is given as $n\epsilon^{n-1}$ by construction, where the prefactor n originates from the number of possible ways to choose $(n-1)$ players who will cooperate by mistake. The probability of transition in the opposite direction is of $O(\epsilon^n)$ at most, but it is reasonable to guess that it also has a prefactor as an increasing function of n. If it can be approximated as $n^\tau \epsilon^n$ with $\tau > 1$, for example, efficiency criterion should require $n \lesssim (1/\epsilon)^{1/(\tau-1)}$. To achieve cooperation among a large number of players with finite error probability, therefore, we may have to revise the rules so as to adjust the prefactors.

#12 In Abstract, we have specified the condition of $n = 2$ needed to prove evolutionary robustness:

> A friendly-rivalry strategy for $n = 2$ inherently has evolutionary robustness in the sense that no mutant strategy has higher fixation probability in this population than that of a neutral mutant. Our evolutionary simulation indeed shows excellent performance of the proposed strategy in a broad range of environmental conditions when $n = 2$ and 3.

Below Eq. (16), we have clearly stated that evolutionary robustness for $n \geq 3$ is not proved yet:

> Differently from the two-person case, however, friendly rivalry itself does not necessarily guarantee evolutionary robustness if $n \geq 3$: Assume that a friendly-rivalry strategy y cannot distinguish a mutant x, whereas x does distinguish y when x forms the majority of the n-person game. If $n = 3$, for example, it means that $s_{yyx} = s_{xyy} = \rho$ whereas $s_{yxx} = s_{xxy} = 0$. Furthermore, if the mutants are efficient among themselves, i.e., $s_{xxx} = \rho$, then its fixation probability will be higher than $1/N$. As of now, we find no reason to rule out the possibility of such a mutant.

Also in Discussion, we write, *"it shows excellent evolutionary performance,"* instead of *"evolutionary robustness,"* in L375.

#13 We have newly drawn Fig. 6 after additional calculations. A related discussion is found from L358 to L371 as follows:

We also tested the performance of CAPRI-n against strategies with the same memory length. An obvious problem is the huge number of possible strategies: Provided that $m = 2n - 1$, the number amounts to $2^{2^{nm}} \sim 10^{19}$ for $n = 2$, which grows to 10^{9864} for $n = 3$. As an alternative to exhaustive enumeration, we calculated fixation probabilities of mutants that are randomly sampled from the set of deterministic strategies with $m = 2n - 1$, with taking CAPRI-n as the resident strategy. The numbers of sampled mutants were 10^9 and 5×10^6 for $n = 2$ and 3, respectively. As shown in Fig. 6, none of them had fixation probability greater than $1/N$, and the tendency was more pronounced in the three-person game than in the two-person case. For comparison, we also tested resident strategies drawn randomly from the same strategy set with $m = 2n - 1$, and a significant fraction of mutants succeeded in fixation with probability higher than $1/N$. Although we have no proof for evolutionary robustness for $n \geq 3$, the numerical result suggested that it would be extremely unlikely to see CAPRI-n invaded by random mutants even if they had the same memory length.

#14 The error probablity is now denoted as ϵ although it was originally e.
